# Mathematical modeling of drying of potato slices in a forced convective dryer based on important parameters

**DOI:** 10.1002/fsn3.258

**Published:** 2015-08-03

**Authors:** Samira Naderinezhad, Nasrin Etesami, Arefe Poormalek Najafabady, Majid Ghasemi Falavarjani

**Affiliations:** ^1^ Department of Chemical EngineeringIsfahan University of TechnologyIsfahan84156‐8311Iran

**Keywords:** Air temperature, forced air convective dryer, mathematical kinetics model, potato slices, sample shape

## Abstract

The effect of air temperature, air velocity, and sample shapes (circle and square with the same cross‐sectional area) on kinetic drying of potato slices in a tunnel dryer was investigated experimentally and a suitable drying model was developed. The experiments of drying of potato slices were conducted at an air temperature of 45–70°C with an air velocity 1.60 and 1.81 m sec^−1^. Results showed that drying temperature was the most effective parameter in the drying rate. The influence of air velocity was more profound in low temperature. The time for drying square slices was lower compared to the circle ones. Furthermore, drying data were fitted to different empirical models. Among the models, Midilli–Kucuk was the best to explain the single layer drying of potato slices. The parameters of this model were determined as functions of air velocity and temperature by multiple regression analysis for circle and square slices. Various statistical parameters were examined for evaluating the model.

## Introduction

Drying of agricultural products is one of the main goals of preservation, transportation, and commerce. Drying process reduces the content of moisture and then chemical alterations help minimizing microbiological activity in the product during the storage. Therefore, the persistence of product and its stability will increase.

The traditional method which is used in drying of fruits and vegetables through sunlight is not common nowadays, it takes more time leading to a low‐quality product with microbial growth and low process capability. Some effective and rapid methods are required to solve these problems in the process of industrial drying. The most common method in drying foodstuffs is the hot air drying which causes rapidity, produces high capacity, reduces its weight and volume, and also minimizes the cost of packing, storage, and transportation. Dried products are affected by drying conditions such as temperature, relative humidity, air velocity, and physical and chemical parameters of raw materials.

An important source of carbohydrate which is rich in *β*‐carotene, fiber, and potassium ion is sweet potato. Potatoes are also the fourth most important vegetable crop for human nutrition in the world. The starch of sweet potato can be used as an ingredient in bread, biscuits, cake, etc.

Dried potato is used to produce potato flakes, powder, animal feed, and chips. Prepared potato's powder is useful for sore salve, concentrate conserves, cake flour, flavor improver, bread paste improver, pizza paste, etc.

One of the important aspects of drying technology is the modeling of drying process. It has been (study of drying behavior of different materials) an interesting subject for many researchers, in both theoretical and application way. Drying kinetics of food is a complex process. To predict the rate of drying and optimization of drying parameters need suitable kinetic models. Many models have been proposed by researchers for kinetics drying of biological materials. Models of the thin layer drying are very common (see Table 1). The process of drying which includes a thin layer of sample particles or slices is called thin‐layer drying.

**Table 1:** Single layer mathematical drying model

No.	Model name	Model	References
1	Page	MR = exp(*−kt* ^*n*^)	Guarte (1996), Zhang and Litchfield (1991)
2	Modified page	MR = exp[*−*(*kt*)^*n*^]	White et al. (1981)
3	Two term	MR *= a*exp(*−k*_0_ *t*) *+ b*exp(*−k*_1_ *t*)	Rahman et al. (1997)
4	Diffusion approach	MR *= a*exp(*−kt*) *+ *(*1 − a*)exp(*−kbt*)	Kassem (1998)
5	Verma et al.	MR *= a*exp(*−kt*) *+ *(*1−a*)exp(*−gt*)	Verma et al. (1985)
6	Midilli–Kucuk	MR *= a*exp(*−kt* ^*n*^) *+ bt*	Midilli et al. (2002)
7	Weibull	MR* = *exp(*−* ((*t/b*)^*a*^))	Marabi et al. (2003)
8	Modified page equation 2	MR = exp(− c* ((*t/*(*b* ^*2*^))^*n*^))	Diamante and Munro (1991)

Hatamipour et al. (2007) analyzed changes in the structure and color of potato samples/slices in different dryers and in different conditions.

Aghbashlo et al. (2009) developed the kinetic model by investigating the thin layer drying kinetics of potato slices in continuous dryer under different air temperatures and air velocity.

Akpinar (2006) examined drying of potato, apple, and pumpkin in the cyclone dryer under forced airflow and estimated kinetic models as a function of sample area, temperature, air velocity, and time for prediction of moisture ratio on trays of dryer. Hassini et al. (2007) measured water loss and volume variation curves for potato samples during drying. They evaluated potato moisture diffusivity from convective drying kinetics with correction for shrinkage and presented correlations for prediction of moisture diffusivity as a function of moisture content and air temperature.

Senadeera et al. (2003) investigated the effect of temperature and samples aspect ratio on drying behavior of parallelepiped shaped potato, cylinder shaped green bean, and sphere shaped pea in fluidized bed dryer and established kinetic models for drying them.

Tripathy and Kumar (2008) investigated the drying process of potato in slice and cylinder shapes in natural convection mixed mode solar dryer. They provided models for each sample and represented temperature which was dependent on drying parameters for cylinders and slices of potato.

Drying of potato with different air temperature, air velocity, and size of the samples was studied experimentally in the cyclone dryer by Akpinar et al. (2003). They developed models as a function of mentioned parameters for trays of dryer.

Reyes et al. (2007) studied the effects of drying conditions such as type of dryer, drying temperature, and air velocity on drying time and the quality of dehydrated potato slices. They also established a model to explain drying curves of potato slices.

Although some of the previous researchers provided general models as a function of parameter that affect drying process (Akpinar et al. 2003; Akpinar 2006; Tripathy and Kumar 2008). While there is not any study about the effect of sample shape on drying rate of potato. In this study, the effects of some important parameters such as air temperature, air velocity, and sample shape on drying behavior of potato in a force convective dryer are experimentally investigated. There is also a general mathematical kinetic model as a function of air temperature and air velocity for two sample shapes with the same surface area which is developed and compared with experimental data.

## Materials and Methods

### Experimental setup

**Figure 1:**
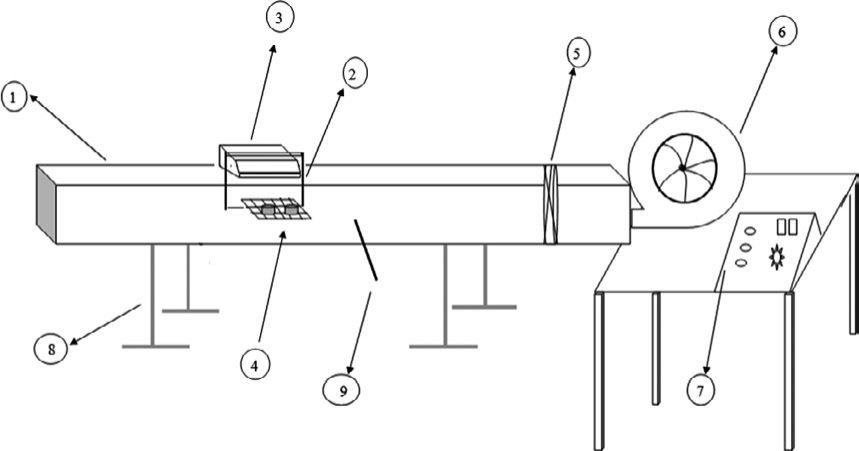
Experimental setup: 1, duct; 2, digital balance; 3, lever; 4, tray and samples; 5, mesh elements of heater; 6, fan; 7, control panel; 8, stand; 9, thermometer.

A schematic diagram of the designed experimental setup has been shown in Figure 1. It includes a blower with 90 m^3^ h^−1^ of nominal flow rate, heater elements with variable power (100–3600 W), and air channel with dimensions 0.15 × 0.15 × 2.43 m^3^ that contains a polymer mesh tray. As a uniform airflow forms near the tray, it has been located 2 m from the entrance of the channel. Air temperature and air velocity are adjusted for the samples on the tray by changing the power of the heater and valve of the blower, respectively. Hot airflows parallel to the tray over and under the samples and the samples are dried in two directions, top and bottom. Before the tray, the air temperature is measured by thermometer with readability of *±*1°C. A digital balance (Shimadzo Co., Nakagyo‐ku, Kyoto, Japan) was used to measure the sample weight on line. Its readability is 0.01 g.

All experiments were carried out in the same environmental conditions (ambient pressure is 610 mmHg and ambient temperature is 26°C). Multifunction measuring instrument (Testo‐400, Lenzkirch, Germany) was used to measure the velocity of airflow. The range of velocity measurement is 0 to10 m sec^−1^ with this instrument and an accuracy of ±0.03 m sec^−1^. Some experiments were repeated three times in the same conditions to check the repeatability of results and the average of standard deviation was 0.0346.

The samples were placed on the tray after reaching to a steady‐state condition for airflow velocity and temperature. The loss of weight is measured by gravimetric method during that time. In order to measure the dry solid of samples, samples were dried in a vacuum oven at 70°C for 24 h.

### Sample preparation

Potatoes used for sample preparation were from the same race, fresh, and uniform. They were kept in dark and cold place (4°C) before the experiments were conducted. The average initial moisture content of the samples was approximately 449.1% (dry weight basis).

Potato samples/slices were cut into circle and square shapes with the same cross‐sectional area (about 20 cm^2^) and thickness (7 mm).

In order to maintain the quality of products, such as color of the sample while drying, pretreatment procedure was done (Akpinar et al. 2003; Agarry et al. 2005; Aghbashlo et al. 2009).

For this purpose, samples were blanched for 5 min in boiling water at 90°C before drying and exactly after the cutting. The time of heating should not be so much that the samples get on the verge of being cooked. Samples were then immediately immerged in 500 mL of cold water at 10°C for 2 min, the water on the surface of the samples was removed by a tissue or filter paper, and finally the samples are ready for drying test.

In order to investigate the effect of various parameters on the drying of potato samples, three series of experiments were designed which are as follows:

1. Experiments with different average velocities (1.6 and 1.81 m sec^−1^).
2. Experiments with different temperatures (45, 50, 55, 60, 65, and 70°C).
3. Experiments with different shape of samples (square and circle with the same cross‐sectional area).

In each series of experiments, other parameters had been fixed.

### Drying data processing

Moisture content (g water/g dry solid) of the sample (M) is calculated by the following equation (1):

(1) $$M=\frac{m_{t}-m_{s}}{m_{s}}$$

where *m*_t_ is the mass of sample at time *t* and *m*_s_ is mass of dry solid.

The moisture content is essential to be the same for all samples in the base measure because of natural and inherent differences in initial moisture content of the samples. Therefore, the moisture ratio (MR) expression is calculated using the following equation:

(2) $$\text{MR}\;=\;\frac{M_{t}-M_{e}}{M_{o}-M_{e}}$$

where *M*_t_, *M*_e_, and *M*_0_ are moisture content at any time, equilibrium moisture content, and initial moisture content, respectively. In this method, samples were placed in an environment maintaining relative humidity and temperature constant. When the change in the weight of samples was insignificant, the moisture of the samples was measured and adopted as the equilibrium moisture content (*M*_e_). In order to low humidity in ambient (dry and hot weather), equilibrium moisture content is negligible and MR* = M*_*t*_ */M*_0_.

Drying curves were constructed by MR versus time. Numerical differentiation of drying curves is proportional with drying rate. This concept is used as dimensionless drying rate (DR) through the paper.

### Mathematical drying model

Various mathematical and semi‐empirical models have been reported in order to study the mathematical modeling of thin layer drying kinetics. In this study, 21 different expressions were examined. Between those expressions, the best eight were selected that can be seen in Table 1.

Nonlinear regression analysis was performed using MATLAB software (version 7.8).

Root mean square error (RMSE) (Procopio (2007)), chi‐square (χ^2^) (Peck and Devore (2011)), *R*‐square (*R* ^2^) (Vidakovic (2011)), and mean absolute error (MAE) (Frees (2010)) were used to determine the quality of the fit. Higher values of *R* ^2^ and lower values of *χ* ^2^, MAE, and RMSE indicate better goodness of fit (Doymaz 2008; Usub et al. 2010). These can be calculated as:

(3) $$\chi^{2}=\frac{\sum_{i=1}^{N}{\left(\text{MRexp},\;i-\text{MRpre},\;i\right)}^{2}}{N-Z}$$

(4) $$\text{RMSE}={\left[\frac{1}{N}\sum_{i=1}^{N}{\left(\text{MRpre},\;i-\text{MRexp},\;i\right)}^{2}\right]}^{1/2}$$

(5) $$R^{2}=1-\frac{\sum_{i=1}^{N}{\left(\text{MRexp},\;i-\text{MRpre},\;i\right)}^{2}}{\sum_{i=1}^{N}{\left(\bar{\text{MRexp}}-\text{MRexp},\;i\right)}^{2}}$$

(6) $$\text{MAE}=\frac{1}{N}\sum_{i=1}^{N}\left|{\text{MR}}_{\mathrm{pre},i}-MR_{\exp,i}\right|$$

where MR_exp,*i*_ is the experimental moisture ratio found in any measurement, MR_pre_,_*i*_ is predicted moisture ratio for this measurement, *N* is the number of observations, *Z* is the number of model constants, $\bar{{\text{MR}}_{\exp}}$ is the total average data, and *i* is *i*th data.

## Results and Discussion

The operating conditions for different sets of the experiments are presented in Table 2. In this section the effect of various parameters on the potato drying process is investigated.

**Table 2:** Operating conditions in different sets of experiments

Parameter	Unit	Value
Velocity	m sec^−1^	1.60, 1.81
Shape	–	Circle, Square
Temperature	°C	45, 55, 60, 65, 70 (circle)50, 60, 70 (square)

Plotted drying curves for all experiments showed that there is no constant rate period in drying of potato slices and falling rate period occurs in drying process of potato due to diffusion controlled mechanism inside the potato. In the early stage of drying process, heat and mass transfer rate is high in thin layer drying. The drying process decreases exponentially and becomes slower and eventually stops by decreasing the gradient of water concentration in the sample. The lack of a constant rate period of drying can be due to changes in the internal moisture and surface changes owing to the shrinkage of the samples during the drying process.

### Effect of air temperature

**Figure 2:**
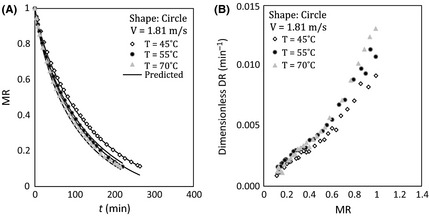
(A) Moisture ratio versus time and (B) dimensionless drying rate versus moisture ratio at different temperatures, 1.81 m sec^−1^ of air velocity, and circle sample.

**Figure 3:**
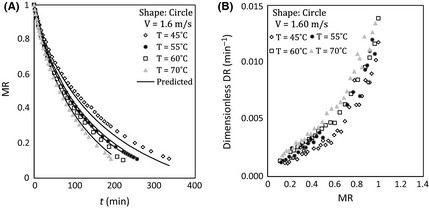
(A) Moisture ratio versus time and (B) dimensionless drying rate versus moisture ratio at different temperatures, 1.60 m sec^−1^ of air velocity, and circle sample.

Figures 2 and 3 indicate the effect of air temperature on dimensionless moisture content and dimensionless drying rate in high and low velocity of airflow for circle sample during drying. Figures 2A and 3A illustrate that total drying time decreases and drying rate increases with temperature. For example, the time of drying reaching MR *= *0.11 was reduced from about 335 to 256 min by changing temperature from 45°C to 55°C for circular sample at 1.60 m sec^−1^ air velocity.

As shown in these figures, the effect of temperature on drying process at first stage is great. This behavior is attributed to the fact that driving force for evaporation of water from the samples increases by heating the sample.

**Figure 4:**
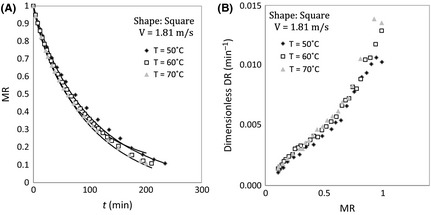
(A) Moisture ratio versus time and (B) dimensionless drying rate versus moisture ratio at different temperatures, 1.81 m sec^−1^ of air velocity, and square sample.

It is also found that the effect of temperature on dimensionless drying rate is more profound in lower air velocity by comparing Figures 2B and 3B. Similar trend was seen for square shape samples for variation dimensionless moisture and dimensionless drying rate as shown in Figure 4.

Similar results can be found from drying curve that have been presented by Aghbashlo et al. (2009) in the length of continuous band dryer. The results show that the increment in drying rate of potato with air temperature in low air velocity is more than that in high air velocity.

The effect of temperature on drying behavior of potato has been investigated by several researchers at various air velocities; in a cyclone dryer at 50, 60, 70°C (Akpinar et al. 2003), in a laboratory scale, convective vertical downward flow dryer at 40, 55, 70, 85°C (Hassini et al. 2007), in fluidized bed dryers at 30, 40, 50°C (Senadeera et al. 2003). Similar findings are reported by them.

### Effect of air velocity

**Figure 5:**
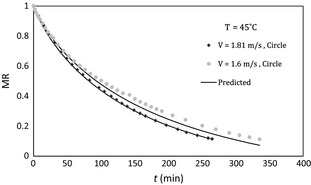
Moisture ratio versus time at different air velocities, 45°C air temperature, and circle sample.

**Figure 6:**
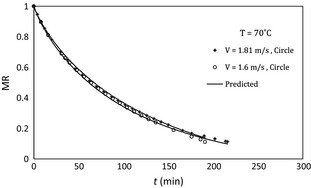
Variation in moisture ratio versus time at different air velocities, 70°C air temperature, and circle sample.

The variation in dimensionless moisture content with time in low and high temperature has been shown in Figures 5 and 6, respectively. It is found that drying rate is increased with air velocity.

The resistance to mass transfer is mixed in the potato samples and in the external layer. Thickness of the boundary layer and thus the mass transfer resistance in the gas phase decreases with blowing airflow on the sample and increases drying rate consequently. Toward the end of the drying, the effect of air velocity would become negligible because of the greater internal resistance to moisture transfer caused in part by structure changes. Similar results can be found in the previous works (Akpinar et al. 2003).

Comparing Figures 5 and 6 show that the influence of air velocity on drying rate accretion in low temperature is more than that in high temperature (square samples showed similar trends). This effect has not been directly discussed in the previous studies, but similar results can be understood from drying curves that are reported in some of the previous works (Hassini et al. 2007; Aghbashlo et al. 2009).

### Effect of sample shape

**Figure 7:**
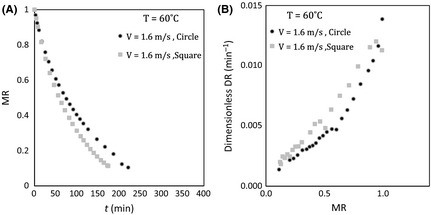
(A) Moisture ratio versus time and (B) dimensionless drying rate versus moisture ratio at different shapes, 60°C air temperature, and 1.60 m sec^−1^ air velocity.

Figure 7 illustrates the variations in dimensionless moisture during drying and dimensionless drying rate against dimensionless moisture for two samples, respectively. The effect of shape parameter (circle and square section area) with the same surface area (=20* *cm^2^) has not been examined in the previous studies. According to the Figure 7A, sample in square shape dried faster than the circle shaped sample. Figure 7B shows that the shape parameter has much influence on drying rate.

Although the cross‐sectional area is considered equal in both samples, lateral surface area of the square sample is more than that in circle sample. This would increase the surface exposed with airflow and increase the evaporation rate consequently. In addition the ratio of perimeter to cross‐sectional area for square shapes is more than circle. This increases the heat transfer and since the temperature in the drying was most effective parameter, mass transfer increases.

### Mathematical modeling

**Figure 8:**
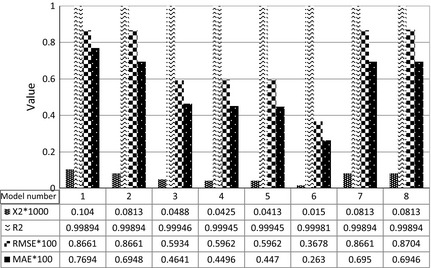
Average of various statistical parameters in comparison with eight of the best model for circle shape tests.

Averages of various statistical parameters were calculated determining the quality of the fit for the best eight models in Table 1 (Fig. 8). As seen in this figure, Midilli–Kucuk model (with the number 6 in Table 1) is the best (parameters calculation were repeated for square samples and similar results obtained).

Several researchers have investigated kinetic drying model of potato. But only some of them (Akpinar et al. 2003; Akpinar 2006) have developed a general model with significant statistical parameters. Nonlinear regression analysis is performed to develop a comprehensive model with goodness of fit in this study.

It is assumed that there is no resistance in airflow to transfer moisture in proposed model. Selected model (Midilli–Kucuk model) is:

(7) $$\text{MR}=a\text{exp}(-k\left(t^{n}\right))\;+\;bt$$

where *a* and *n* are dimensionless, dimension *k* is (time^*−n*^), and dimension *b* is (time^−1^).

Coefficients of the selected model have been calculated in Table 3 for all tests. Statistical parameters have been calculated to compare the goodness of fit by equations (3) to (6) and they have been listed in this table. According to Table 3, high value of average *R* ^2^ (0.99973) and low value of average *χ* ^*2*^ (0.000025) indicate that fit is good.

**Table 3:** Midilli–Kucuk coefficient (eq. (7))

Test no.	*V*m sec^−1^	*T*°C	Shape	*a*	*b*	*n*	*K*	*R* ^2^	*χ* ^2^	RMSE	MAE
1	1.81	45	Circle	1.00900	0.00019‐	0.87280	0.01404	0.99986	0.00001	0.00299	0.00249
2	1.81	55	Circle	1.00700	−0.00031	0.85460	0.01722	0.99990	0.00001	0.00268	0.00222
3	1.81	60	Circle	1.00800	−0.00019	0.82340	0.01873	0.99991	0.00001	0.00245	0.00182
4	1.81	70	Circle	1.00800	−0.00019	0.84520	0.02004	0.99986	0.00001	0.00323	0.00247
5	1.6	45	Circle	1.00600	−0.00020	0.79850	0.01658	0.99979	0.00002	0.00385	0.00268
6	1.6	55	Circle	1.00800	−0.00018	0.84670	0.01680	0.99988	0.00001	0.00345	0.00288
7	1.6	60	Circle	1.00100	−0.00024	0.86600	0.01596	0.99937	0.00005	0.00694	0.0042
8	1.6	65	Circle	1.00700	−0.00036	0.84050	0.01806	0.99988	0.00001	0.00285	0.00231
9	1.6	70	Circle	1.00800	−0.00038	0.83610	0.02042	0.99982	0.00001	0.00383	0.00228
10	1.81	50	Square	0.99020	−0.00003	0.94170	0.01207	0.99927	0.00008	0.00892	0.00759
11	1.81	60	Square	1.00400	−0.00025	0.86840	0.01775	0.99983	0.00001	0.00350	0.00248
12	1.81	70	Square	1.00200	−0.00017	0.86070	0.01981	0.99974	0.00002	0.00447	0.00376
13	1.6	50	Square	0.99520	−0.00005	0.93500	0.01173	0.99928	0.00008	0.00869	0.00729
14	1.6	60	Square	1.00100	−0.00019	0.94360	0.01451	0.99972	0.00002	0.00475	0.00397
15Extra run	1.34	50	Square	0.99720	−0.00008	0.99900	0.00911	0.99983	0.00002	0.00393	0.00322

In order to generalize the drying kinetic model, coefficients of Midilli–Kucuk expression were modeled with various parameters such as temperature, air velocity, and sample shape by multiple regression technique. Some researchers have done this for potato, in the past (Akpinar et al. 2003; Akpinar 2006; Aghbashlo et al. 2009; Tripathy and Kumar 2008).

In addition to air temperature and air velocity, the shape of samples is considered in the model too. Coefficients of Midilli–Kucuk model as the best fitting model involves the drying variable such as temperature, air velocity for two shapes of potato sample were determined as follows:

a=1.00389

$$b\;=\;\frac{\alpha +V}{\beta +\gamma T}+\varphi$$

$$n\;=\;\tau \text{exp}\left(\omega T\right)\times V^{\theta }$$

$$k\;=\;\rho {\left(\text{TV}\right)}^{\sigma }$$

where α, β, γ, φ, τ, ω, θ, ρ, σ are constants and they are determined in Table 4.

**Table 4:** Constant parameter of presented models (Midilli–Kucuk kinetic model)

Shape	α	*β*	*γ*	φ	*τ*	*ω*	Θ	*ρ*	*σ*
Circle	−1.80211	2.24510E+4	−3.04645E+2	−0.00021	0.7859	0.00019	0.11191	0.00137	0.55637
Square	0.00859	−3.23366E+10	6.46733E+8	−0.00020	1.29904	−0.00339	−0.29405	0.00005	1.25228

Validation of these three functions is evaluated with %E (Mean of relative error percent), RMSE, and *χ* ^2^. Low values of %E, RMSE, and *χ* ^2^ show good fit of Midilli coefficients model with presented model. These values are reported in Table 5.

**Table 5:** Validation of Midilli–Kucuk coefficient model with statistical parameters

Shape	Coefficient	% *E*	*χ* ^*2*^	RMSE
Circle	*b*	15.73	4.6E‐09	0.00005
	*n*	1.90	0.00061	0.02022
	*k*	5.17	1.58E‐06	0.00111
Square	*b*	11.11	3.39E‐09	0.00003
	*n*	1.81	0.00086	0.01858
	*k*	6.63	2.51E‐06	0.00123

By comparing computed dimensionless moisture contents with measured ones, the validation of final established model for drying kinetic of potato with coefficients as the function of process parameters was made in all drying runs (14 runs). *R* ^2^ and slopes have been provided for all runs for measured MR versus predicted MR in Table 6. A good agreement between model and experiment data has been shown in Table 6. It also can be seen that the drying model has successfully predicted the drying of potato in Figures 2A, 3A, 4A, 5, and 6.

**Table 6:** Slope and *R* ^2^ for predicted MR versus experimental MR

Test no.	*R* ^2^	Slope
1	0.9998	0.9835
2	0.9998	0.9697
3	0.9996	1.0522
4	0.9998	1.0114
5	0.9997	1.0565
6	0.9996	0.9899
7	0.9993	0.9762
8	0.9999	0.9775
9	0.9996	0.978
10	0.9989	1.0069
11	0.9993	1.029
12	0.9949	0.9089
13	0.9978	1.0248
14	0.9996	0.9859
15Extra run	0.9991	1.0218

The derived model (by 14 runs) is examined by test 15 as extra run; air velocity 1.34 m sec^−1^ with 50°C air temperature and square shape sample; that it was not included in the multiple regressions. The results confirm that present model is suitable for tests which are out‐of‐range for multiple regression tests.

## Conclusion

Drying process of potato slices was investigated in hot air convective dryer. No constant drying rate period was observed. The effect of air temperature, air velocity, and sample shape of potato slices were examined on behavior of potato drying. Results showed that air temperature has the most effect on decreasing of drying time. The influence of air velocity on drying rate in low temperature is more than that in high temperature due to the dominant temperature effect. The time of drying for square slices of potato is lower than circle ones under the same test conditions according to greater lateral surface area. A suitable drying model was developed by incorporating the effect of above parameters on potato drying process. The regression coefficients and statistical parameters RMSE, *χ* ^2^, and MAE values show that the derived model is a good agreement with data and it would be a useful engineering application such as design and optimization.

## Conflict of Interest

None declared.
